# Big Data in Israeli healthcare: hopes and challenges report of an international workshop

**DOI:** 10.1186/s13584-015-0057-0

**Published:** 2015-12-07

**Authors:** Christian Lovis, Ronni Gamzu

**Affiliations:** Division of Medical Information Sciences, University Hospitals of Geneva and University of Geneva, 4, rue Gabrielle-Perret-Gentil, Geneva, 1205 Switzerland; Tel Aviv Sourasky Medical Center, Tel Aviv, Israel

## Abstract

In September 2015, the Israel National Institute for Health Policy Research (NIHP) organized a workshop to address the hopes and challenges of Big Data in healthcare in the Israeli context. The paper provides an overview of the challenges and hopes raised by data driven science and Big Data, along with a summary of Israel’s strengths and weaknesses regarding Big Data, as discussed by the speakers in the course of the conference. It concludes with some hints on how Israel’s advantages in this field might be leveraged.

## Background

In September 2015, the Israel National Institute for Health Policy Research (NIHP) organized a workshop to address the hopes and challenges of Big Data in healthcare in the Israeli context. The workshop, chaired by Ronni Gamzu, was a unique occasion to allow fruitful discussions between decision makers, researchers, and care providers, and prominent national and international experts in the field. The participants represented most sectors of health, governmental agencies, and industry.

**Fig. 1:**
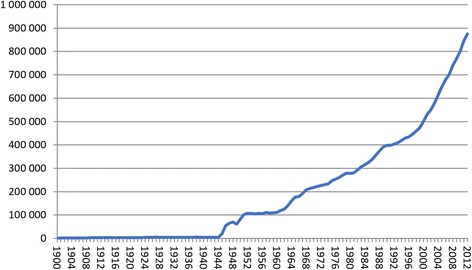
Yearly citation count from 1900–2012 MEDLINE/PubMed Baseline: 21,927,394 citations

This paper reports the personal view and understandings of the authors—the conference chairperson, and one of the conference’s invited speakers from abroad. The intention of this paper is to present a short overview of Big Data in health and its challenges, as well as some ideas for the future. It is also an attempt to provide an external insight on the situation of Big Data in Israel and action plans that should be undertaken.

### Big Data in health

According to the Health 2020 report of WHO [1], healthcare systems in the 21st century are on an unsustainable course. Maximizing health through all stages of life is critical, and good health is an asset and a source of economic and social stability. Good health is a global capital resource and can no longer be seen as the outcome of one sector alone. Sustainable health is the product of numerous determinants across all parts of life (including the environment, sanitation, exposure factors, and lifestyle), as well as collaborative efforts across all parts of society. In order to progress towards a better understanding of health determinants, triggers of diseases and innovative approaches for prevention and care, new paradigms for research and policy makers are needed. Data driven science and data driven decision making is one of the most promising way to achieve it [2]. It has already demonstrated a significant impact on providers’ behaviour [3].

The Feb 2010 edition of “The Economist” introduces the concept of “The Data Deluge” [4]. The resulting exponential growth in data quantities far outpaces the capability of human being to cope, so that the data are largely unexploited. This is true for almost all aspects of human activities, such as cell phones activity, mobility, finance and economy, supply chains, environmental data, etc. It is true in health when it comes to data about patients and citizens, such as electronic health records (EHR) or the quantified self, biobanks and genomics, images, etc. It is also true in the knowledge produced by humans, and especially in medicine. The number of scientific publication indexed in PubMed exceeded 22 million at the end of 2014 [5] (Fig. 1) and continues to grow at a rapid pace.

Big Data is expected to provide significant benefits for individuals, such as in personalized medicine, but also for healthcare systems. The next generation of healthcare systems will need to exploit the ever-increasing quantities of complex, massively multivariate data concerning all aspects of health determinants, patient care, and outcomes that are starting to be routinely acquired and stored, throughout the life of each individuals of a population. In 2005, in response to its earlier findings in To Err is Human [6] and Crossing the Quality Chasm [7], the Institute of Medicine (IOM) introduced the concept of Learning Healthcare System. This concept has subsequently evolved into a broader concept of a learning health system, as an ecosystem where all stakeholders can securely, effectively and efficiently contribute, share and use data to create knowledge and support effective decision-making, leading to improved health outcomes.

There are a growing number of examples of citizens and patients starting to benefits from personalized medicine, such as in pharmacogenomics [8]. The hopes and promises to improve health and care outcomes significantly by allowing the development of citizen- and patient-specific probabilistic health models open the door to personalised medicine.

Data integration across spatial scales, from the molecular to the population level, and across temporal scales, from genomics to lifestyle, is one of the key challenges for exploiting these massive, disparate datasets in health.

The definitions and realities of Big Data are still the object of discussions and debates. Big Data is a broad term that designates datasets usually characterized by their large size and high complexity, thus challenging traditional data analytics. All aspects of data management are involved, from data acquisition and capture, through the stages of storage, transfer, access and sharing, models for data representation and semantic of the data, curation or quality management, analytics or learning, visualization or human interactions, interpretation and finally the capacity to support decisions (which includes reliability and accuracy).

The Big Data’s term originates in 2001 with the three Vs defined by Doug Laney [9]: Volume, Velocity, and Variety. In his original vision, Doug Laney considered:

(V)olume for very large size;
(V)elocity for high bandwidth and I/O throughput;
(V)ariety for numerous datatypes and semantics.

Later, the (V)elocity also included real time analysis of streams of data, such as real-time analytics on social networks activity. Slowly, up to eleven V were further added, such as:

4. (V)alue: Data is becoming the new currency in the economy. The vast amount of data available is used to do targeted marketing, individual pricing, deep learning, personalisation, predictive analytics, amongst others;
5. (V)ariability: flows can be highly inconsistent with periodic peaks;
6. (V)eracity: the reliability of data.

### Dimensions

It is difficult to find metrics to characterize Big Data properly, and there is no clearly defined ways of addressing this point. However, in a non-exhaustive approach, five dimensions can be used:

- Extent, relates to the number of individuals, the number of sources, the number of elements considered. Interoperability, privacy, or how data can be linked are among the challenges raised by the extent of elements;
- Deepness or granularity, represents how detailed are the data considered, how much information is there for each element. Representing data, models or semantic are among the challenges raised;
- Density is the space between elements, or the amount of links between elements or their data;
- Temporal length is duration considered. It is one of the most difficult challenges. Big Data analytics handles large volumes of data generated in short time, but also have to handle data generated in a long period. In the latter case, the problems of handling the temporal variability of the semantics, the evolution of the dataset, of external factors influencing the data, etc. become crucial;
- Interpretability is the fact that the data can be used, and how much, for the required purpose, sometimes named “actionable data” in analogy to genomics. This is finally the real value of the data and the question the “autonomy” of the dataset: How far are all information and knowledge required to “action” the data available. In addition, to what extend shall all of it be available on a computerized format.

### Challenges

In order to gain all the potential hidden in the massive amounts of data available, there are numerous challenges to address. They involve much more than the challenges inherent in the usual data management and analytic field; they raise important policy and regulatory issues, with complex societal, ethical, technical and scientific dimensions.

### Privacy

One of the interesting aspects of Big Data is to connect data from numerous sources. It can therefore create a kind of virtual image of a person. This characteristic is already being exploited for many widespread purposes such as targeted marketing, personalized pricing, risk evaluation for insurers, etc. It is much more complex in health, but it is the goal of most ongoing initiatives in the field. However, when it comes to health, building trust is an essential aspect and it implies to provide citizens with confidence in the whole system. Trust must be built at all levels, from technical levels, such as security, to the governance and transparency of the usage of the data, such as consent management. The recently published report “*The Precision Medicine Initiative Cohort Program*” by the U.S. National Institute of Health, promotes a very transparent, open, dynamic and proactive vision of consent [10]. However, it must be kept in mind that the data available on individuals are increasingly able to unleash all aspects of life, including political, religious, racial, and behavioural and lifestyle determinants, and raise new threats that will require new responses.

### Regulatory and policy

Regulatory aspects have to deal with many aspects of Big Data, at the fine edge between providing an enabling environment, while keeping a legal framework able to build trust and protect the interests of all stakeholders. Finding a good balance between protection and enabling is a difficult challenge. However, there are many aspects where the government can provide significant inputs. The field of Big Data is recent, and there is a strong and major shortage of experts in the field, of education paths, of research and development, of outcome analysis. All of these should be supported massively, and promptly. Interoperability of data is a major challenge, especially in health, which can only be addressed by fast development and adoption of standards, especially in the field of semantics. Numerous data sources are playing critical role in health, such as studies raw data, drug data, but also mobility, environment, exposition factors, etc. While these data often exist, they are rarely available, and enforcing open data can leverage access to this information.

### Quality

Data quality is a recurrent problem in data management. However, quality management takes on a new face in the Big Data era. Numerous approaches and methods to improve data quality have been used and sometimes misused, in traditional data management and with Big Data [11]. One of the interesting feature of having numerous or streamed sources in Big Data is the frequent impossibility to improve data quality at the source, such as when dealing with information written in social networks. Another example is dealing with data quality over a long period of capture or in distributed sources, when there are variations of the quality in time and by source. One of the most promising approaches is to replace data curation by a continuous description of data quality, which itself becomes a new data source.

### Probabilistic decision support

Deep learning is one of the techniques allowing analytics on large and unstructured data sources. However, this type of approach, like many others, requires having a well-balanced source of information. For example, when building automatic learning using the medical literature, it becomes crucial that all negative results be published and rewarded equivalently to positive results. Otherwise, probabilistic learning and thus decision support based on large corpuses of published papers will be systematically biased. New ways of presenting results will have to be developed, such as description of the coverage of the data sources, its reliability, etc. in order to be able to evaluate the probability that the answers are correct and evaluate the predictive value of the results.

### Distributed analytics

One of the characteristic of health data is that it is usually difficult to build fully centralized repositories. This is partly due to legal and regulatory frameworks, partly due to the volume of some datasets, such as medical images; and finally due to the complexity and highly variable of some structured datasets, such as formularies. This aspect of health data is also emphasized in the *One million cohort project* which recommends a hybrid data and analytics architecture that leverages both centralized data storage of core data while preserving federated access to additional data [10]. However, distributed storage implies also distributed analytics, and thus developing new robust, reliable, and reproducible methodologies to evaluate correlation and causality.

### Data streams and complexity

Increasingly, the data will be in streams (the river of data) rather than datasets (the lake of data). Most of the traditional biostatistics and epidemiological analytics rely on analyses of datasets, rather than analysing flows of data in real-time. Complex probabilistic models, real-time analytics such as bio-surveillance networks, raise new challenges, as has been well shown with Google Flu Trends [12, 13]. Good evaluation of these new models is necessary, and, most probably, they will leverage traditional models rather than replace them.

### The hypothetic-deductive approach

The hypothetic-deductive approach is the traditional approach in epidemiology and clinical research: a hypothesis is raised; it leads to a study design that tries to minimize bias and confounding while maximizing generalizability, a sample size calculation is carried out to ensure enough power, and the results are allowed to infirm or confirm the hypothesis. A second approach, data mining, extends this approach at, usually, analysing datasets to see if “something is in”, sometimes named “fishing” the data. Big Data, when considered over long period of time, introduces a third approach: how is it possible to represent and store data and metadata in order to allow the dataset to answer, in the future, questions not known when the data were created and collected. This is similar to long-term cohorts follow-up: how to design the data management so that it will be usable for future questions. The major challenge here is to enrich the metadata and use very flexible models of representation.

### “Unstructured” data

Increasing the amount of structured data is one of the leitmotivs often heard when using EHRs because unstructured data is considered as not usable for clean analytics. In health, unstructured data is mostly made of free text and narratives, then images and signals. However, considering these sources as unstructured is fallacious. These sources are, in fact, highly structured. They are only poorly analysable with computers. Human languages are highly evolved and structured medium of communication. In addition, they convey very dense and rich information, and usually represent most of the information available. Thus, they must be considered as such, and important efforts invested in order to improve the capacities to analyse them, such as natural language processing, image recognition, etc.

### Big Data and Israel

Throughout the workshop, it was very clear that there are very important assets in Israel, especially when it comes to data in the healthcare sector. The data—“Big Data”—are available! A small number (four) of large HMOs cover all the population, and hospitals as well are consolidated in two main groups: the governmental hospitals (approximately half) and those owed by Clalit HMO (almost a third). In addition, HMO’s and hospitals have a long history of computerized records. From the dimensions of Big Data discussed above, this asset is one of the richest in the world in five dimensions: length, deepness, density and temporal length. The latter is a precious advantage: More than 20 years of history of capturing health data by most stakeholder is a precious asset. A lot more can and should be done with it [14].

However, having the data is not enough if it is not made actionable, and the challenges discussed above must be addressed. A priority to address is interoperability, and very strong efforts should be undertaken in order to promote the development and the adoption of interoperability standards, especially for semantics and data models. This goes along with the need to develop competences in the complete chain from data generation, to capture, and encoding, to analytics and finally to the use of this asset in order to leverage the healthcare system and promote health. This will only be possible with strong interventions mixing regulatory framework, sustained support and incentives.

While there are top scientists, start-ups and SME’s in the field of Big Data in Israel, there is a lack of global awareness and competencies in the many other fields involved. Introduction of the topic in the curriculum of care professionals, managers, decision makers to name a few is still marginal. There are only very few pre- and post-graduate specific education paths. This lack of global expertise could produce a massive problem in a near future taken into account the speed of the evolution of the field.

Table 1 summarizes these, and additional, strengths and weaknesses that Israel brings to the field of Big Data.

**Table 1:** Big Data: Israel’s strengths and weaknesses

Strengths
1. Strong high tech industry; out-of-box thinking
2. Expertise in natural language systems
3. Health plan databases
a. All Israeli residents are members of a health plan
b. Large number of members in each plan
c. Unique patient IDs
d. Diverse population
e. The rate of transferring among plans is very low
f. Health plans have many years of longitudinal data
g. All primary care physicians and most other providers have electronic health records
h. Health plans integrate high quality data from a large number of providers (including direct care professionals, labs, pharmacies, etc.) and do so almost in real time
i. Growing national HIE system, integrating all health plans and all hospitals
4. Health plans know how to use their databases for care improvement
5. Health plans have a strong motivation to make care more efficient
6. Limited number of health care system actors and less fragmentation; makes coordination easier
Weaknesses
1. Storage and computational capacity in the health plans is large, but not as large as in Google, IBM, etc.
2. Lack of guidelines on what health data may be put on the cloud
3. Relative long negotiation times for high tech-health plan collaborations
4. Lack of clarity regarding who owns the data
5. Shortage of funds for evaluation studies
6. Not getting enough input from patients on the Big Data initiatives
7. Professionals and organizations who are very protective of their data
8. Insufficient awareness of the potential of Big Data to improve care and make it more efficient
9. Health plans viewing their data as an asset to be monetized
10. Health plan data systems very focused on clinical data; only now beginning to pull in data on patient experience, patient preferences and patient-reported outcomes
11. Slow-moving IRB processes

## Conclusion

Israel’s current unique advantages might not be so unique in a few years, when the new global players in the field, and especially large companies such as Amazon, Apple, Google or IBM, will team up in more favourable environments. This can be in a context less constrained with regulatory frameworks, or better incentivized, or with improved penetration of massive data production tools, such as quantified-self, bio-banking, etc. These challenges can only be addressed if the philosophy in the healthcare system will move from a competitive approach to a cooperative and collaborative approach. This is true not only for public-private partnerships, but also between academic centres or care providers. The Big Data area is multidisciplinary in its essence, and it is wide by nature. It requires converging efforts.
